# The effects of methylcellulose coating containing carvacrol or menthol on the physicochemical, mechanical, and antimicrobial activity of polyethylene films

**DOI:** 10.1002/fsn3.2240

**Published:** 2021-03-18

**Authors:** Elahe Jahdkaran, Seyed Ebrahim Hosseini, Abdorreza Mohammadi Nafchi, Leila Nouri

**Affiliations:** ^1^ Department of Food Science and Technology, Science and Research Branch Islamic Azad University Tehran Iran; ^2^ Food Science and Technology Department Damghan Branch Islamic Azad University Damghan Iran; ^3^ Food Biopolymer Research Group Food Technology Division School of Industrial Technology Universiti Sains Malaysia Minden Penang Malaysia

**Keywords:** active packaging, antimicrobial properties, edible coating, essential oil

## Abstract

In this study, the physicochemical, mechanical, and antimicrobial activities of polyethylene (PE) films coated with peppermint (Menthol) and *Origanum vulgare* (Carvacrol) essential oil were evaluated. For this reason, PE films were coated with MC‐HPMC solution containing different concentrations of menthol and carvacrol (0, 1, 1.5, and 2%), and mechanical, electromagnetic, barrier, and antimicrobial properties of all prepared films were examined. The obtained results demonstrated that by increasing the concentration of menthol and carvacrol in film coatings, tensile strength (from 36 to 23 MPa), water vapor permeability (from 12 to 11 g.m^‐1^s^‐1^Pa^‐1^), and L* and b* indexes were decreased, while the oxygen permeability (OP) and elongation at break significantly were increased (*p* < .05). Increment of menthol and carvacrol concentration in PE film coating leads to an increase in the antimicrobial activity of films against *Escherichia coli*, *Staphylococcus aureus*, *Listeria innocua,* and *Saccharomyces cervicea*. Finally, the results obtained from this study demonstrated that PE film coated with high levels of carvacrol and menthol could be used as active antimicrobial packaging in the food packaging industry.

## Graphical abstract

Carvacrol and menthol were used as two natural antimicrobial agents in active packaging. PE films by coating is an effective method to making active packaging.

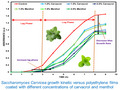

## 1 INTRODUCTION

Today, the demand for food products and food packaging is increasing as the world's population grows. Food packaging can protect the foods packed in it against various environmental factors such as light, oxygen, pressure, heat, and water vapor (Fattahi et al., 2020; Mousavian et al., 2021; Nouri & Mohammadi Nafchi, 2014; Tavakoli et al., 2019). Packaging can also protect the food products against destructive chemical reactions and microbiological contamination and improve the shelf life of products by increasing their safety. Recently, new packaging saves have been used to maintain food products quality, that active and intelligent packaging is one of the most popular packaging (Sharma et al., 2020; Vinh et al., 2020; Xue Mei et al., 2020).

Active packaging is a new and interesting packaging in the food industry that increases the shelf life of packed products by increasing food safety and maintaining their quality during the storage period (Hosseini et al., 2021; Kazemi et al., 2020; Vilela et al., 2018; Wang et al., 2020). These active systems often contain active compounds that can be released into the interior of packaging. This type of packaging system usually uses active ingredients derived from natural sources that have antioxidant and/or antimicrobial activities (Lukic et al., 2020; Moslehi et al., 2021). However, in order to prepare active packing from synthetic polymers, bioactive compounds cannot be used directly in the production stages of these polymers because the performance of these active agents is reduced due to the high temperatures used for processing. One of the methods used for this problem often involves the use of carriers for bioactive agents, which through these carriers, bioactive compounds can be placed on the surface of synthetic polymers (Jafarzadeh et al., 2020; Nor Adilah et al., 2020; Rahmani et al., 2017).

In recent years, much attention has been paid to the isolation and use of new bioactive compounds with antioxidant and/or antimicrobial activities from plant sources (Daneshzadeh et al., 2020; Sharma et al., 2020; Tavakoli et al., 2017). Carvacrol (2‐methyl‐5‐(1‐methylethyl)‐phenol) is a monoterpenic phenolic bioactive compound isolated from aromatic plants and some essential oils such as *Origanum*, *Thymus vulgaris,* and *Carum copticum* (Jeon et al., 2018). Carvacrol has shown significant antimicrobial activities against the range of gram‐positive bacteria, gram‐negative bacteria, and fungi (Dhumal et al., 2019). For example, in a study conducted by Nostro and Papalia (2012), the antimicrobial activity of carvacrol against a wide range of food pathogens and spoilage microorganisms was investigated and confirmed. The researchers found that the antimicrobial activity of carvacrol is due to its effects on the structure and function of the microbial cytoplasmic membranes.

Menthol is cyclic terpene alcohol extracted from plant sources that have been used for various medicinal purposes (A Farco & Grundmann, 2013). This bioactive compound is abundant in the essential oil of the peppermint plant. Various scientific studies have shown that menthol has antifungal and antimicrobial activity (Trombetta et al., 2005; Turcheniuk et al., 2015). Researchers have shown that the antimicrobial activity of menthol is probably due to its effects on microbial membrane permeability, which leads to the leakage of intracellular compounds (Kamatou et al., 2013).

Due to the increasing demand of consumers to increase the consumption of natural preservatives to extend the shelf life and develop the safety of food products, in this study, carvacrol and menthol were used as two natural antimicrobial agents in active packaging. Since these compounds are heat sensitive, they have been used to coating PE films, and their effects on the basic and antimicrobial properties of PE films were investigated.

## 2 MATERIALS AND METHODS

### 2.1 Materials

Polyethylene (PE) film was prepared from Sultan Chap Co. (Karaj, Iran), with thicknesses of 0.02 ± 5.7 × 10^–4^ (mm). Carvacrol and menthol (CAS 64‐17‐5 EMSURE^®^ Reag. Ph Eur) and Mueller Hinton Broth (MHB) (110,293—Merck Millipore) were purchased from the Merck (Darmstadt, Germany). *E. coli* O157:H7, *S*. *aureus* ATCC 6,538*, Listeria innocua, and Saccharomyces cervicea* were also collected from the Institute of Science Research and Technology (Tehran, Iran). All other chemicals used were of analytical grade.

### 2.2 Preparation of PE films coated with carvacrol and menthol

A coating solution was prepared from methylcellulose (MC) and hydroxypropyl methylcellulose (HPMC). 7 g MC and 3 g HPMC were slowly added to 200 cc of 95% ethanol and heated on a hot plate with a magnetic stirrer. When the temperature reached 65ᵒC, heating was stopped. Simultaneously with stirring, the mixture of polyethylene glycol and 100 cc of distilled water, as a plasticizer, was added to the cooled mixture of MC‐HPMC (Rardniyom, 2009). Then, carvacrol and menthol were added to the coating solution at different concentrations so that the coating materials containing antimicrobial agents were obtained at the final levels of 0, 1.0, 1.5, and 2% w/w. The resulting coating solution was applied to the PE film using a roller, and then, the films were dried under normal conditions (Cooksey, 2005). Each of the antimicrobial agents used, including carvacrol and menthol, were applied separately to the PE film. The addition of antimicrobial agents and coating of films prepared at low temperatures was done to minimize the loss of antimicrobial agents due to their volatility.

### 2.3 Characterization of PE films coated with carvacrol and menthol

#### 2.3.1 Measurement of thickness

The thickness of the films was measured by a digital micrometer with an accuracy of 0.001 mm. Measurements were made at five points on the films, and their average was used in calculations related to physical properties tests such as water vapor permeability.

#### 2.3.2 Evaluation of mechanical properties

In order to investigate the mechanical properties of PE film samples based on ASTM Standard Method E96‐05 (ASTM, 2005) with some modification, first, the film strips were cut to a length of 10 mm and a width of 20 mm and was exposed to ambient temperature and relative humidity of about 50% for 2 days. Then, a texture analyzer (TA.XT2, Stable Micro System, Surrey, UK) was used to measure the mechanical properties of the film (tensile strength and elongation at break). The initial velocity separation and cross‐head were selected as 50 mm and 30 mm/min, respectively, and the tensile strength at the point of rupture from the deformation and the force recorded by the software were calculated.

#### 2.3.3 Evaluation of heat‐sealing capability

In order to evaluate the sealing capability of the films, heat sealability was measured according ASTM‐F88 method (ASTM, 2015). Two layers of film (2 cm) were placed on top of each other and sewn together by a thermal sewing machine with a temperature of 10 ᵒC and the same sewing time. A stripe of films measuring 1 and 4 cm was prepared. While the distance between the two jaws of the machine was defined as 20 mm, the trip was stretched at a speed of 10 mm/min until the film was torn, and the sealing strength was measured by a texture analyzer and was reported in Newton per meter (N/m).

#### 2.3.4 Evaluation of water vapor permeability (WVP)

Water vapor permeability was measured according to ASTM Standard E96/E96 M‐16 (ASTM, 2016) by a desiccator containing magnesium nitrate supersaturated solution. The test method was that small glass vials were selected, and 3 g of anhydride calcium chloride was poured into each vial, and the glass surface was covered with films and clamps. Due to the absorbent moisture of anhydride calcium chloride, the relative humidity of the interior of the glass vial and the below the film was zero percent. After initial weight, all samples were transferred simultaneously to a desiccator containing magnesium nitrate salt, which generates 55% humidity at laboratory temperature. Changes in the weight of glassware over time were measured using a digital scale to accurately 0.001 g, and the graph of weight changes versus time was drawn, and the slope of the resulting line was used for calculations.

#### 2.3.5 Evaluation of oxygen permeability (OP)

In order to investigate the oxygen permeability of PE films, the ASTM Standard Method D3985‐17 and a Mocon Oxtran 2/21 system (Minneapolis, USA) were used (ASTM, 2017, 1996). Film samples were conditioned at a temperature of 25ᵒC, atmospheric pressure, and relative humidity of 55% for 48 hr, and then, their thickness was measured and mounted into the diffusion cell of the equipment. By using the convergent method, OP of the films was estimated by WinPermTM permeability software.

#### 2.3.6 Determine color indexes

A colorimeter (Minolta CM‐3500D; Minolta Co. Ltd., Osaka, Japan) was used to evaluate the color indexes of PE film sample. Before measuring the color of films, the device was adjusted using a standard white screen. The color parameters of device are the intensity of lightness or L* index (white = 100 and black = 0), the intensity of red and green or a* index (green= −80 and red = 100), and the intensity of yellow and blue or b* index (blue= −80 and yellow = 70. At least three points were measured in each film sample (Arezoo et al., 2020).

#### 2.3.7 Examination of the UV‐Visible spectra

The inhibitory properties of PE films against UV and visible light were studied by measuring their light absorption by spectrophotometer UV/Vis at wavelengths of 200 to 800 nm (Pereda et al., 2011). The films were cut into rectangles (1 × 4 cm) and placed directly on the machine cell. Air was used as a reference.

#### 2.3.8 Investigation of microbial growth kinetics

Investigation of microbial growth kinetics of *Escherichia coli*, *Staphylococcus aureus*, *Listeria innocua,* and *Saccharomyces cervicea* against PE films was performed using the dynamic test (shake flask method) by expressed by Sadeghnejad et al., (2014). In this study, bacteria grown in 100 ml of Mueller Hinton Broth (for one night) were used. 2 × 10^5^ CFU/mL of each bacterium was added to 100 ml of Mueller Hinton Broth and incubated for 12 hr at 37 ᵒC. Every 2 hr, the absorbance changes were read by spectrophotometer UV/Vis at a wavelength of 600 nm, and bacterial growth was obtained. All steps of the test were repeated 3 times, and the mean values were used.

### 2.4 Statistical analysis

Statistical analysis of data was performed using IBM SPSS Statistics 22.0 (SPSS, Inc.). One‐way analysis of variance (One‐way ANOVA) following by Duncan's multi‐range test used to compare means at 5% significance level among different samples.

## 3 RESULTS AND DISCUSSIONS

### 3.1 Effects of coating PE film with carvacrol and menthol on the mechanical properties

**FIGURE 1:**
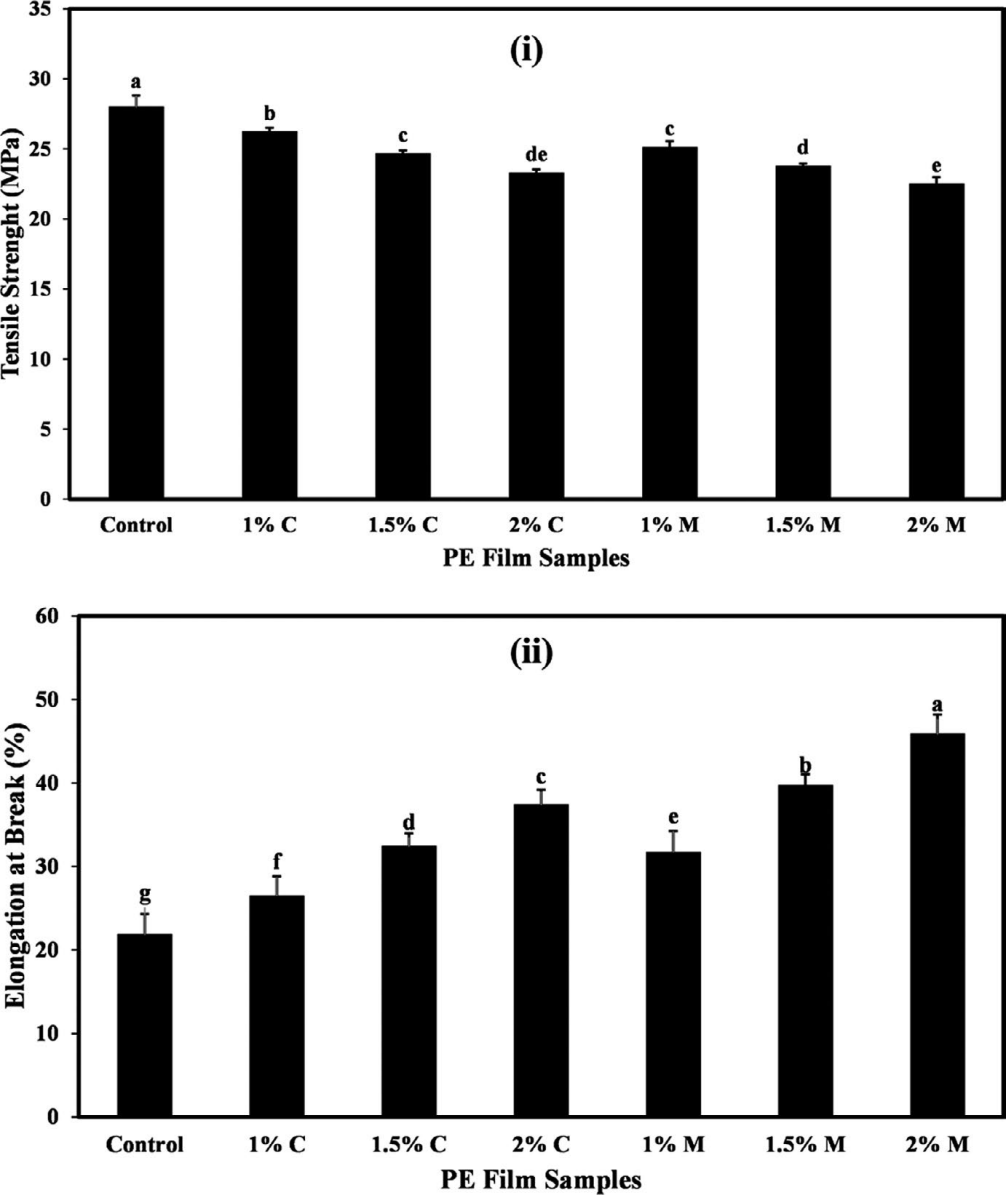
(a) The tensile strength values (MPa) and (b) elongation at break (%) of PE films coated with different levels of carvacrol and menthol. Bars represent mean (*n* = 10) ± *SD*. Different letters on the bars indicate a significant difference at 5% level of probability among PE films. C: Carvacrol, M: Menthol

The average values of the tensile strength (ST) and elongation at break (EB) of different coated PE films are shown in Figure 1a, b, respectively. As the results show (Figure 1a), the highest ST was related to the control film (27.95 MPa), and coating of PE films with different levels of carvacrol and menthol led to a significant reduction in the ST of produced films (*p* <.05). So that the lowest amount of ST was obtained in films containing the highest level of carvacrol and menthol (2% level) (23.24 and 22.46 MPa, respectively).

The results of the EB amounts of the coated PE films (Figure 1b) also indicated that by coating the PE films with different levels of carvacrol and menthol, a significant increase in the EB of produced films was observed (*p* <.05). Thus, the control had the least amount of EB (21.80%), and with increasing the concentration of carvacrol and menthol in the coating, the amount of this mechanical index increased significantly (*p* <.05). The highest amount of EB is related to the film coated with a solution containing 2% menthol (45.80%).

Mechanical properties (such as tensile strength and elongation at break) are important factors in food packaging. Tensile strength indicates the maximum tensile force that the film can withstand, and the amount of elongation at break indicates the maximum change in the length of the test sample before breaking (Pereda et al., 2011). The mechanical properties of films are influenced by several factors, including the interactions between film compositions, temperature, physical, and chemical conditions (Sánchez‐González et al., 2011). The change in the uniformity of the film network structure due to hydrophobic properties of carvacrol and menthol is the reason for the decrease in tensile strength and the increase in elongation percentage of films containing high levels of these active compounds.

Based on research conducted by various researchers, the addition of high levels of antimicrobial compounds to various polymers due to changes in molecular level changes the mechanical properties of films produced from these polymers. These changes cause the destruction of the film network and, in most cases, act to reduce the resistance of the films to stretching (Chen et al., 2017, 1996). Rubilar et al., (2013) also agreed with the results of the present study that by adding high levels of carvacrol and grape seed extract to the chitosan film, the values of the tensile strength of film samples were significantly reduced (*p* <.05). Jutaporn et al., (2011) also showed that with increasing Phayom wood extract in films, tensile strength and elongation percentage were reduced. Rojas‐Grau et al., (2007) observed that with increasing concentration of lemon essential oil (from 0.05% to 1%), the tensile strength of films decreased, which was consistent with the results of the present study. However, the addition of cinnamon essential oil at similar levels reduced the strength of the films. These researchers said that the difference in the performance of different compounds was due to differences in the polarity of added compounds. Another study also reported that the addition of black pepper and ginger essential oils to composite films increased tensile strength and reduced flexibility of film (Amalraj et al., 2020).

### 3.2 Effects of coating PE film with carvacrol and menthol on the heat‐sealing strength

**FIGURE 2:**
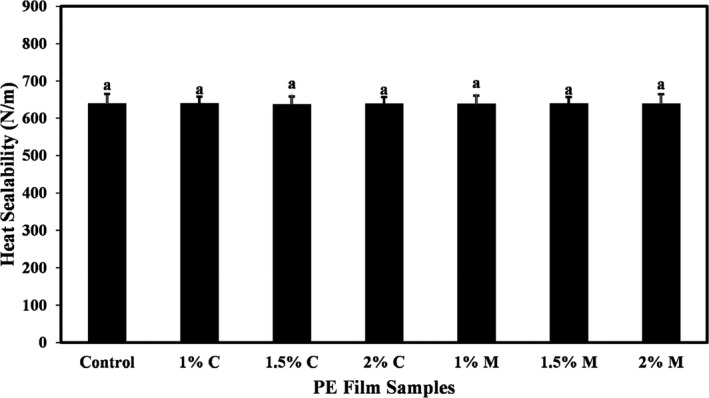
The heat‐sealing strength (N/m) of PE films coated with different levels of carvacrol and menthol. Bars represent mean (*n* = 10) ± *SD*. Different letters on the bars indicate a significant difference at 5% level of probability among PE films. C: Carvacrol, M: Menthol

The values of the heat‐sealing strength of PE films coated with different concentrations of carvacrol and menthol are summarized in Figure 2. As can be seen in Figure 2, the use of coatings containing different levels of carvacrol and menthol did not have a statistically significant effect on the heat‐sealing strength of PE film. The average values of the heat‐sealing strength of PE films ranged of 636.36 to 638.96 N/m.

In general, the heat‐sealing capability of a material is important for its use in the food packaging industry. Heat sealing is the process of sealing a flexible film to another similar film under heat and pressure. The quality of heat sealing depends on process conditions such as sealing methods, temperature, pressure, time (sealing time and cooling time), type of film materials, and treatments. Regardless of the various parameters for the quality of heat sealing, the resulting seal must be strong enough to maintain the integrity of the package. This seal must have high mechanical strength to be able to protect the packaged products (López et al., 2011).

### 3.3 Effects of coating PE film with carvacrol and menthol on the water vapor permeability (WVP) of films

**FIGURE 3:**
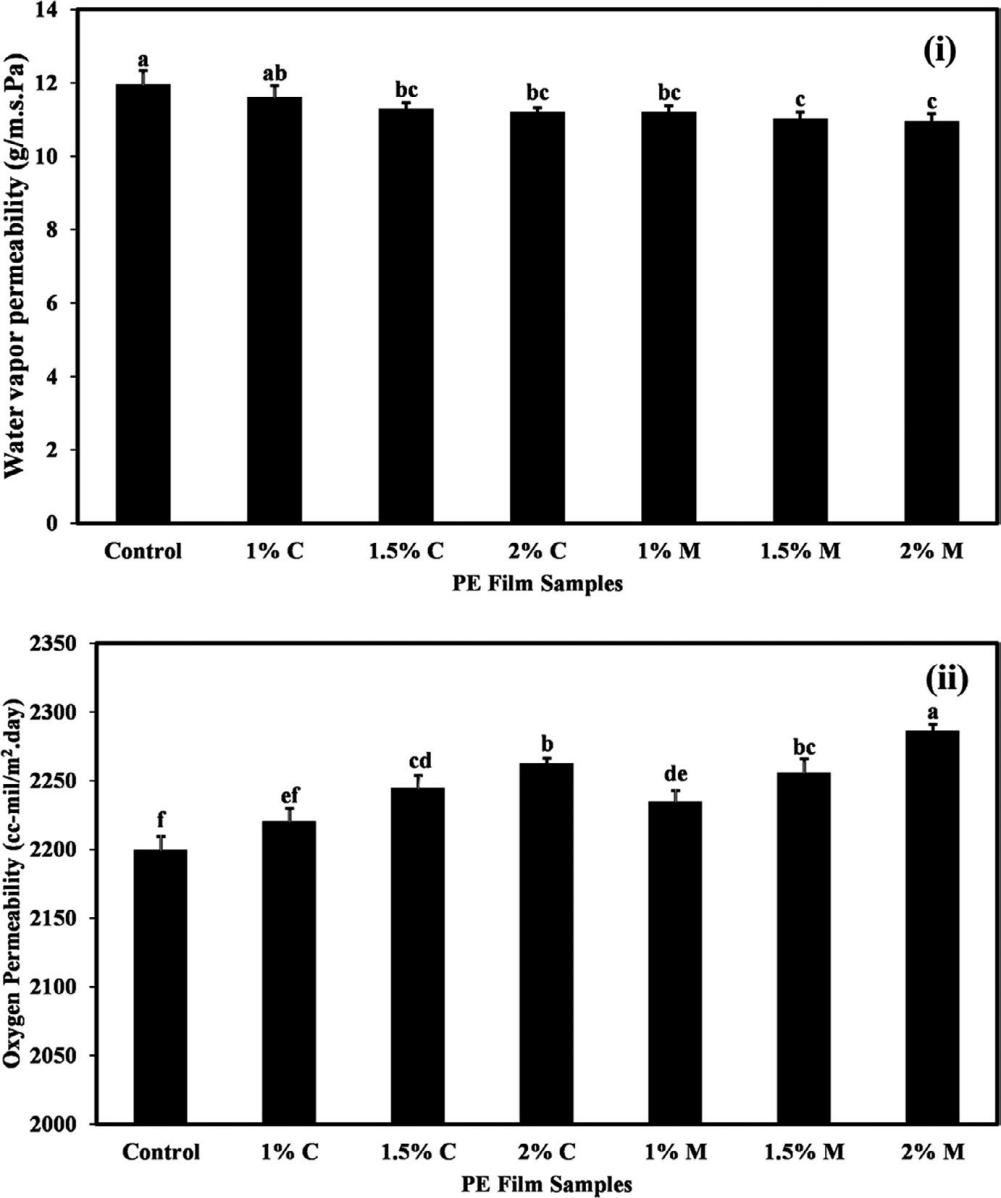
(a) The WVP values (g/m.s. Pa) and (b) oxygen permeability values (cc‐mil/m^2^.day) of PE films coated with different levels of carvacrol and menthol. Bars represent mean (*n* = 10) ± *SD*. Different letters on the bars indicate a significant difference at 5% level of probability among PE films. C: Carvacrol, M: Menthol

Figure 3a compares the average values of WVP of PE films coated with different concentrations of carvacrol and menthol. It shows that the highest WVP amount was obtained in the control film (11.93 g/msPa), and coating of the PE film with different levels of carvacrol and menthol reduced the WVP amounts of the films (*p* <.05). However, increasing the concentration of these additives in the coating solution did not show a significant effect on WVP of the film.

One of the most important characteristics of food packaging is its permeability to water vapor, which is due to the important role of water in destructive reactions and the growth of microorganisms. Water acts as a solvent or carrier and causes texture decomposition and chemical and enzymatic reactions. High relative humidity is one of the main causes of food spoilage. Therefore, the water vapor barrier is one of the important properties of polymers used for food packaging.

Water vapor transfer is generally done through the hydrophilic part of the film and therefore depends on the ratio of hydrophilicity to the hydrophobicity of film compounds (Giteru et al., 2015). Various factors such as the ratio of hydrophilic to hydrophobic groups and curvature and fracture in the film structure affect the amount of water vapor permeability (Wong et al., 1992). The reduction in water vapor permeability of PE films, likely related to the hydrophobicity nature of carvacrol and menthol.

Some researchers believe that incorporating hydrophobic active compounds (such as essential oils) into polymer films can reduce the rate of water transfer by reducing the effect of plasticizers (Giteru et al., 2015). Rubilar et al., (2013) also stated that increasing carvacrol levels in chitosan film samples significantly reduced the WVP of films. Jutaporn et al., (2011) similarly observed that with increasing the concentration of Phayom wood extract in the film, the WVP increased significantly, which was due to structural changes in the polymer film. Decreased WVP rate of sago starch‐guar gum due to the incorporation of carvacrol and citral in the active films was also reported by Dhumal et al., (2019). In a study of the effect of clove essential oil on carboxymethylcellulose (CMC)‐based films, Dashipour et al., (2014) showed that the WVP decreased with increasing concentration of clove essential oil in films.

### 3.4 Effects of coating PE film with carvacrol and menthol on the oxygen permeability (OP) of films

Oxygen permeability of packaging materials plays an important role in food preservation because oxygen is a key factor in oxidation reactions and several other destructive reactions (Sothornvit & Pitak, 2007). The permeability of films to oxygen depends on the crystallinity of the films so that as the crystallinity increases, the permeability of films to gasses (oxygen and carbon dioxide) decreases (Miller & Krochta, 1997). Figure 3b shows the values of OP of different PE films. The results demonstrated that coating PE film with different levels of carvacrol and menthol led to an increase in the OP of films, and with increasing the level of these additives in samples, a significant increase in the OP values of films was observed (*p* <.05). The OP value in the control sample was 2,199 cc‐mil/m^2^.day and in the films coated with the highest levels of carvacrol and menthol increased by 2,262 and 2,286 cc‐mil/m^2^.day, respectively. This increase in permeability due to the use of carvacrol and menthol in the PE film coating is probably due to the change in the structure of the films and the increase in the holes in them, which increases the permeability of the films to oxygen. Altiok et al., (2010) also observed an increase in oxygen permeability due to the addition of thyme essential oil to the chitosan film. In another study, an increase in the oxygen permeability of edible films was reported due to the addition of high levels of grape seed extract and carvacrol (Rubilar et al., 2013).

### 3.5 Effects of coating PE film with carvacrol and menthol on the color indexes of films

The color of films used in food packaging is very important because of their effects on food consumer acceptance. The color indexes of different samples of PE coated with different levels of carvacrol and menthol were measured by the Hunterlab, and the values of the indexes obtained are given in Table 1. The results showed that the highest L* index was related to the control film (68.71), and the coating of PE film with different concentrations of carvacrol and menthol caused a significant decrease in the L* index (*p* <.05). The lowest L* value was obtained in film coated with a solution containing 2% carvacrol (57.07), but there was not a statistically significant difference between this film and films coated with 1.5% and 2% menthol. Decreased brightness of PE films due to the use of carvacrol and menthol coatings is due to the color of these compounds. On the other hand, due to the hydrophobicity of carvacrol and menthol, probably due to the use of these compounds, the moisture content of the film is reduced, and the color brightness is also reduced.

**TABLE 1:** The color indexes of PE films coated with different levels of carvacrol and menthol

Film samples	L*	a*	b*
Control	68.71 ± 0.42 a	10.35 ± 0.14 a	6.74 ± 0.15 a
1% C	62.69 ± 0.44 b	10.29 ± 0.13 a	6.40 ± 0.10 b
1.5% C	59.65 ± 0.38 d	10.44 ± 0.11 a	6.15 ± 0.13 cd
2% C	57.07 ± 0.41 e	10.49 ± 0.12 a	5.82 ± 0.10 e
1% M	60.57 ± 0.46 c	10.44 ± 0.08 a	6.29 ± 0.11 bc
1.5% M	57.23 ± 0.39 e	10.50 ± 0.11 a	5.97 ± 0.15 de
2% M	57.71 ± 0.25 e	10.37 ± 0.13 a	5.88 ± 0.14 de

Note

Values represent mean (*n* = 5) ± *SD*. Different letters in each column represent a significant difference at 5% level of probability among sample films. C: Carvacrol, M: Menthol.

However, coating of PE films with a solution containing different levels of carvacrol and menthol did not show a significant effect on the a* color index, and the values of this color index were in the range of 10.29 to 10.50. In terms of the b* color index, coating of PE films with different concentrations of carvacrol and menthol led to a significant reduction in this color index (*p* <.05), so that the highest value of b* index was observed in the control sample (6.74) and the lowest amounts were related to the films coated with 2% carvacrol (5.82).

Dashipour et al., (2014) stated that the CMC film is completely transparent and colorless, and the addition of clove essential oil to this film reduced the lightness of the produced films, and the a* index decreased, but the b* color index increased. Pastor et al., (2010) also found that the addition of propolis extract to the hydroxypropyl methylcellulose (HPMC) reduced the brightness of films. The decreased light intensity of sago starch‐guar gum films due to the adding of carvacrol and citral in these films was also reported by Dhumal, Ahmed, et al., (2019).

### 3.6 Effects of coating PE film with carvacrol and menthol on the light transmission of films

**FIGURE 4:**
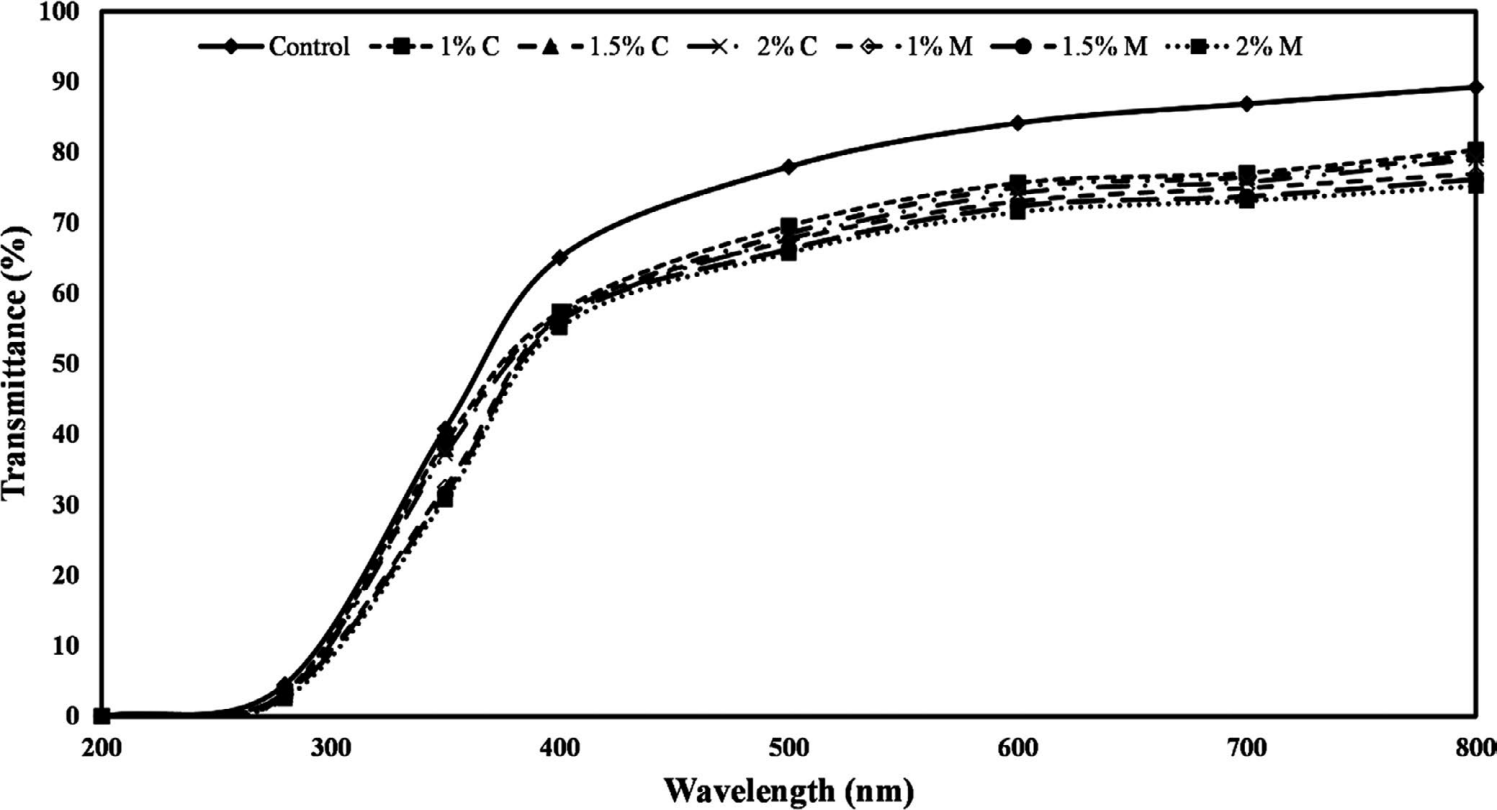
Percentage of light transmission (%) of PE films coated with different levels of carvacrol and menthol. Bars represent mean (*n* = 10) ± *SD*. Different letters on the bars indicate a significant difference at 5% level of probability among PE films. C: Carvacrol, M: Menthol

Figure 4 shows the amounts of UV‐Visible spectra passing through the PE films coated with different concentrations of carvacrol and menthol. The light transmittance of PE films in the wavelength range of 200–800 nm was in the range of 0.005% to 89.22%. At all wavelengths studied in this study (visible and ultraviolet), the coating of PE films with different levels of carvacrol and menthol reduced the amount of light transmission. These results indicate that carvacrol and menthol block the passage of visible and UV light from the film to the product. With the increasing concentration of these two additives, the amount of light transmission gradually decreased.

The optical properties of polymers are related to the degree of crystallinity and their main structure. Decreased transparency of polymers is achieved as a result of reflection, absorption, and scattering of light. The reflection index, reflection coefficient, and scattering coefficient affect the transparency of the film and the amount of light transmission. Therefore, the degree of crystallinity, crystal size, molecular weight, and molecular structure plays an important role in the optical properties of polymers (Ghadermazi et al., 2019). Lopez‐Mata et al., (2013) also observed that adding carvacrol to chitosan film reduced UV light transmission. Tongnuanchan et al., (2014) added basil and lemon essential oils to the gelatin edible films and found that control sample and films containing essential oils did not absorb at 200 nm. However, the light absorption of films containing essential oils at wavelengths less than 280 nm was much lower than the control. The researchers also reported that combining essential oils into films significantly reduced the percentage of light transmission.

### 3.7 Effects of coating PE film with carvacrol and menthol on the microbial growth kinetics

**FIGURE 5:**
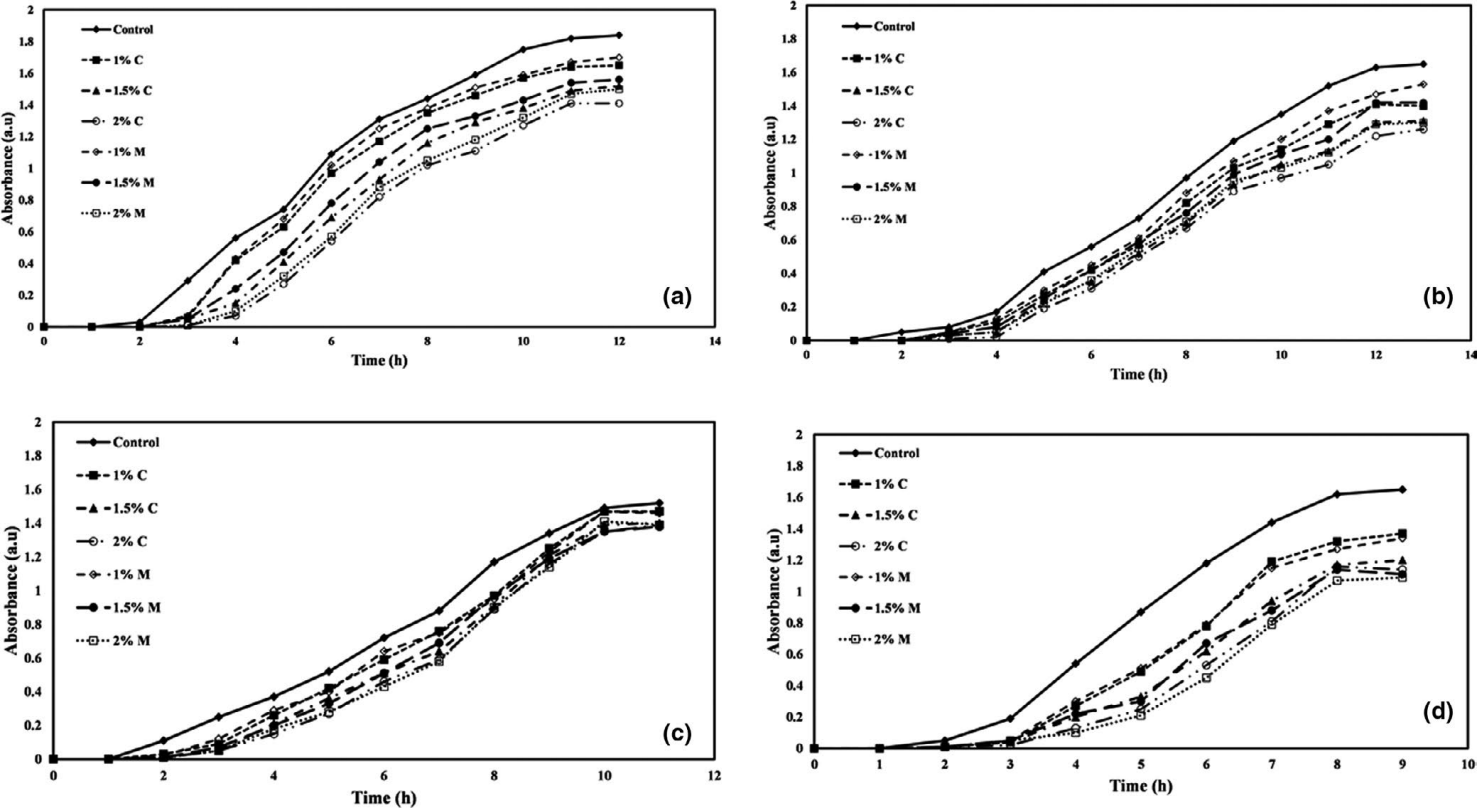
(a) *Escherichia*. *coli*, (b) *Staphylococcus aureus*, (c) *Listeria innocua*, and (d) *Saccharomyces Cervicea* growth kinetic versus PE films coated with different concentrations of carvacrol and menthol. C: Carvacrol, M: Menthol

The microbial growth curve has four phases, including the lag phase, log phase, fixed growth phase, and death phase. In the lag phase, the cell adapts to the new environment and does not increase in number. In the log phase, microorganisms consume food, and their number increases exponentially and logarithmically. In the stage of constant growth, the rate of production is equal to the rate of death, and in the last stage, the number of microorganisms is declining. This stage is also called the self‐destructive phase. If a compound has antimicrobial properties, it can both delay the lag phase and reduce the maximum population of microorganisms in the log phase. The microbial growth kinetics of *E. coli*, *Staphylococcus aureus*, *Listeria innocua,* and *Saccharomyces cervicea* against PE films coated with different levels of carvacrol and menthol are shown in Figure 5a–d, respectively.

The results demonstrated that with increasing the concentration of carvacrol and menthol in the coating, microbial growth kinetics decreased. The lower the microbial growth kinetics, the greater the inhabitation, as the lag phase increases and the log phase decreases. In other words, the active compounds (carvacrol and menthol) kill microorganisms by slowing down the growth rate and prolonging the lag phase of microorganisms or inactivating them. According to the obtained results, the highest level of inhabitation against various microorganisms studied in this study was related to films coated with 2% carvacrol and 2% menthol.

In general, essential oils and plant extracts with hydrophobic compounds can interact with the lipid structure of bacterial, mitochondrial cell membranes, and other intracellular compounds, causing cell structure destruction, ion exchange, preventing respiration and increase permeability, leakage of intracellular compounds, and bacterial cell death (Devi et al., 2010). The antimicrobial mechanisms of action of carvacrol have been previously investigated and found to be due to the interaction of hydrophobic compounds with bacterial membranes. This interference causes changes in the permeability of H^+^ and K^+^ and causes the destruction of basic functions and cell death (Ultee et al., 2002). A gradual release of active molecules into food packaged in active packaging allows these active ingredients to always be available and exert their antimicrobial activity effect in the long run (Amalraj et al., 2020; Xu et al., 2018).

Olasupo et al., (2003) reported that the minimum concentration of carvacrol required to inhibit the growth of *E. coli* (1.5 mmol/L) was greater than the concentration required to inhibit the growth of *Salmonella* (1 mmol/L), and this indicates that *Salmonella* is more sensitive to carvacrol than *E. coli*. Yuan et al., (2015) observed that the chitosan film had no antimicrobial activity against *Staphylococcus,* and with the addition of carvacrol and pomegranate peel extract, the antimicrobial activity against this bacterium increased, and the inhibitory effect of carvacrol on *Staphylococcus* was significantly greater than that of pomegranate peel extract.

## 4 CONCLUSION

Polyethylene film was coated with active antimicrobial compounds of peppermint extract (menthol) and thyme extract (carvacrol). The use of carvacrol and menthol in the coating solution used to coat PE film improved the flexibility and water vapor permeability properties of produced films. Due to the coating of PE films with these active compounds, the barrier properties of the films against the UV‐Visible spectra transmission were improved. Coated PE films with high concentrations of carvacrol and menthol showed good antimicrobial activity against microorganisms studied in this research. In general, the results obtained in this study indicate that PE film coated with carvacrol and menthol can be used as an active food packaging with significant antimicrobial activity in the food packaging industry. In order to accurately understand the effect of carvacrol and menthol on the antimicrobial activity of PE films, further research is needed to investigate their release rate at different times and under different conditions.

## CONFLICT OF INTEREST

The authors declare no conflict of interest.

## ETHICAL APPROVAL

This study does not involve any human or animal testing.
